# Feedback using an ePortfolio for medicine long cases: quality not quantity

**DOI:** 10.1186/s12909-016-0801-3

**Published:** 2016-10-21

**Authors:** Jane Bleasel, Annette Burgess, Ruth Weeks, Inam Haq

**Affiliations:** 1 Education Office, Sydney Medical School, University of Sydney, Sydney, NSW 2006 Australia; 2 Sydney eLearning, Office of the DVC (Education), University of Sydney, Sydney, NSW 2006 Australia; 3 The University of Sydney, Edward Ford Building, Rm 208E, A27, Sydney, NSW 2006 Australia

**Keywords:** e-portfolio, Feedback, Medical students, Clinical long case, Formative assessment

## Abstract

**Background:**

The evidence for the positive impact of an electronic Portfolio (ePortfolio) on feedback in medicine is mixed. An ePortfolio for medical long cases in a Graduate Medical Program was developed. The purpose of this study was to explore the perceptions of medical students and faculty of the impact of the ePortfolio on the feedback process.

**Methods:**

In total, 130 Year 3 medical students, and six faculty participated in the study. This is a mixed methods study, using a combination of both quantitative and qualitative approaches. Quantitative methods were used to quantify the number of long cases performed. Qualitative methods were used to explore the relationship between quantity and quality of feedback, and provide a rich understanding of both students’ and faculty’s experience and perceptions of the ePortfolio.

**Results:**

Students received a variable quantity of feedback at each of the three studied clinical schools, with an average of between 4 – 5.4 feedback episodes per student. Feedback that was constructive, specific and timely and delivered by a senior academic was important. Quantity was not an essential factor, with two episodes of detailed feedback reported to be adequate. The barriers to the use of the ePortfolio were technical aspects of the platform that interfered with student engagement.

**Conclusions:**

Feedback using the ePortfolio for medical long cases is a valuable tool providing a senior clinician delivers detailed, constructive and personalized feedback in a timely fashion. The ePortfolio system needs to be user-friendly to engage students.

**Electronic supplementary material:**

The online version of this article (doi:10.1186/s12909-016-0801-3) contains supplementary material, which is available to authorized users.

## Background

Portfolios are one of many tools used in the assessment of students and provide a potentially useful means of providing timely and detailed feedback. They have been increasingly used in medical education and are emerging as a tool for documenting learning and competencies [1]. In Australian medical schools and in the post-graduate training programs, portfolios are not yet compulsory, although a recent Review of Medical Intern Training has recommended their introduction which is likely to impact on medical schools [2]. The literature, however, suggests mixed success in the use of portfolios in health professional education and feedback from mentors appears to be a crucial contributor to success or failure [1, 3, 4].

### Development of an ePortfolio

In 2015, limited use of an ePortfolio was introduced into the Sydney Medical Program (SMP), a four-year graduate degree at The University of Sydney. The reason for this was to improve the assessment and feedback provided to students in their medical terms. Another driver was to avoid cumbersome paper files in each of the clinical school. In years three and four of the medical degree, students are based predominantly at one of seven clinical schools for terms in Medicine and Surgery as well as a number of specialty blocks. In Medicine they are attached to a medical team with a specified supervisor and the expectation is that they will be involved in all aspects of clinical work, clerking patients and following them through their admission.

Prior to 2015, students were expected to present one case history (long case) per week to their supervisor during an eight week term as part of a formative assessment. There was no formal requirement of students to submit evidence of presenting long cases during the medical blocks and in an end of year survey of students in 2013, 40 % reported completing 1 – 3 long cases [5] whereas they were expected to have completed eight cases.

Feedback for medical students frequently occurs in the clinical setting, and is thus more informal and less predictable than that occurring in the traditional academic setting [4]. A common complaint from medical students and residents is lack of feedback [6]. In keeping with these observations, evaluation of students in 2014 in their third year of the Sydney Medical Program, found that only 35 % of students agreed that they received helpful feedback about their progress [7].

To address these deficiencies, at the commencement of the 2015 academic year, a required formative assessment was introduced. To sit the summative barrier examination at the end of year 3, students were required to submit evidence of completing eight long cases in their medical block via an ePortfolio system. The assessor completed a criterion-based form, with immediate feedback to the student on their verbal presentation. The student was then required to write up the case, having reflected on the feedback, in a concise way, as they would for a medical admission in a patient’s hospital record. The written case and the feedback form were then uploaded onto an ePortfolio system. A Medical Lead at the students’ clinical school also gave feedback on the written, submitted case electronically. Thus students received feedback on their verbal presentation by their supervisor (or other consultant or resident staff) and by another physician, known as a “medical lead”, on their written case. Students had the option of reflecting on the quality, complexity and presentation of the written case.

The ePortfolio system used was Pebblepad, a proprietary, web-based ePortfolio system supported by e-learning at the University of Sydney [8].

The rationale for this study was that feedback provided using the ePortfolio would improve student engagement and enhance perceived competency at this task, secondly that perceived benefit would be related to quantity of feedback. It was also considered essential to describe challenges and barriers to the development of an ePortfolio. The reason for describing the negative aspects of the system was the concern that the time required using the ePortfolio system would negatively impact on the beneficial aspects of feedback.

The aims of this study were to describe students’ experience of using the ePortfolio, and receiving feedback on written long cases; medical leads’ experience of providing feedback on written long cases, and their perceptions of the value of the learning activity for students; and to explore the relationship between quantity and quality of feedback.

## Methods

This is a mixed methods study, using a combination of both quantitative and qualitative approaches. Quantitative methods were used to quantify the number of longcases performed. Qualitative methods were used to explore the relationship between quantity and quality of feedback, and provide a rich understanding of both students’ and medical leads’ experience and perceptions of the ePortfolio.

### Description of setting and educational practice

There are seven broad clinical schools to which medical students are assigned, six metropolitan schools and a rural clinical school (four separate sites). Three of the Clinical Schools; coded as A, B and C, were the settings of the study. All are in the Sydney metropolitan region. Participants in the study included 40 students at Clinical School A, 42 students at Clinical School B, and 48 students at Clinical School C.

### Data collection and analysis

Mixed methods were used to collect and analyse data. Data collection was broken into four categories:

1. The number of completed long cases uploaded to the e-portfolio and episodes of feedback provided
  The software teaching interface provided data on who had given feedback and how many instances. Descriptive statistics were used to describe the number of episodes of feedback provided and person providing feedback.
2. Students’ perceptions of the process of performing long cases, submitting them and receiving feedback
  An invitation to participate in a focus group was extended via email to all year 3 students attending Clinical Schools A, B and C (*n* = 130). Convenience sampling was then used to select 8 participants for one focus group at each of the Clinical Schools (A, B, C). The strategy was to take the first 8 students who responded to the invitation. Student focus groups were conducted by an independent researcher who was not involved with the e-portfolio system (see Additional file 1 for student interview guide). Data were transcribed verbatim, with each participant being assigned an anonymous identifier. The identifier of A, B or C was allocated according to the clinical school of the study participant, followed by a unique number. Thematic analysis was used to build an understanding of the students’ experience. A portion of the data was read by the first author and analysed to identify initial themes. Following negotiation of meanings with the second and third authors, a coding framework was developed and applied to the full data set [9].
3. Medical leads’ experience of providing feedback on written long cases and their perception of the value of the learning activity for students
  Following completion of the student focus group data collection, a convenience sample was used to invite two medical leads at each Clinical School (A, B and C) to individual interviews. Medical Lead interviews were carried out by an independent researcher who was not involved with the e-portfolio (see Additional file 2 for medical leads interview guide). Data were transcribed verbatim, with each participant being assigned an anonymous identifier. The identifier of A, B or C was allocated according to the clinical school of the study participant, followed by a unique number. Thematic analysis of the qualitative data was conducted. While this was initially done inductively by three authors, in subsequent analysis of data, we noted that emergent themes from the inductive analysis resonated with the key themes found in student data. At this point the authors discussed the value of using a similar framework, which was applied to a portion of the data to ensure its consistency, and to check for any new and emerging issues that would extend the analysis. Subsequently, the second author coded all of the data to identify recurrent themes and subthemes in the data.
4. Independent review and analysis of the feedback content provided by the medical leads
  Written feedback provided by medical leads on the long cases was randomly sampled from the three clinical schools, until saturation of themes was achieved. Thematic analysis was used to code and categorise data into themes.

## Results

All Year 3 medical students from Clinical Schools A, B and C (*n* = 130) participated in the study. Fifty five percent of the students were male, and 45 % female.

### The number of completed long cases uploaded to the e-portfolio and episodes of feedback provided

The details of the numbers of students at the clinical schools and total number of feedback episodes (FE) are provided in Table 1. There were between 40–48 students at each clinical school and the average total number of FE was 191 per clinical school. Each student received between 4 – 5.4 FE for his or her eight cases. Clinical School A had the largest percentage of feedback provided by a clinical academic from the Education Office, 83 % compared with 58 % at clinical school C. The aim was that students would receive at least 1 FE for each of the 4 week terms of medicine in a timely fashion to be of most value to the students. The FE were assessed at the completion of the medical term. In Clinical school A, 100 % of students had achieved this feedback whereas at clinical school B and C, the percentage of students who had received timely feedback reduced to 68 and 46 % respectively.

**Table 1:** Details of Clinical school student numbers and episodes of feedback

Clinical School	No of Students	Total No. of FE	Average No. of FE/students	% FE provided by academic medical lead
A	40	216	5.4	83
B	42	175	4.2	60
C	48	182	4	58

*FE* feedback episodes

### Students’ perceptions of the process of performing long cases, submitting them and receiving feedback

Three focus groups made up of 21 students (8 females, 13 males) were conducted.

Three major themes were identified from analysis of the transcripts of the focus groups:

Authenticity of the activity; Feedback and personal development; and Technology and process. Results from the focus groups are illustrated in Table 2. Importantly, students recognized the benefits of practicing and improving competencies relevant to their future careers; and students appreciated the quality and of the feedback, finding that more than two iterations of feedback was not justified. Additionally, students felt reassured that feedback was provided by faculty members, with knowledge of their summative assessment requirements.

**Table 2:** Students’ perceptions of their experience of receiving feedback on written long cases, and the use of the eportfolio

Authenticity of the activity	
Students valued tasks that reflected what they would be doing as interns and beyond. They recognised the benefit of practicing and improving competencies required in their future careers.	T*here was a bit of time pressure and it* r*eflected something that eventually we’ll be doing in the real world…. the real world reality is that you’d have something and then quickly have to present it to someone more senior and it felt like you were actually practising that”. (A 6)* *“I think a more realistic approach would be to have something written up that was just what you wrote down on the wards as – as interns, we’re going to be going to ED, writing down this case, presenting it to a consultant straight up and that’s going to be in the medical records forever…. I’ve seen a lot of bad notes written up and I’m like well I don’t want to be this person. (B2)*
Feedback and personal development	
Quality of the feedback Students appreciated the specificity of the feedback. Students were surprised by the detail and effort by the medical leads in providing feedback. They verbalised a desire for critique of their work rather than non-specific, generic comments. Quantity of feedback Students valued the depth and quality of the feedback and most students felt the incremental benefit from receiving written feedback on more than two of the 8 cases was not justified. Personalised feedback Students appreciated the personal nature of the feedback. Source of feedback Receiving written feedback helped students feel supported by Faculty. Students felt reassured that feedback was provided by a faculty member, familiar with the SMP assessment requirements, and it was important to students that feedback be aligned with summative assessment expectations. Personal development Students commented on the value of the feedback in improving and changing what they submitted. The type of feedback was also important, aiming at producing reflective practice. The students’ approach was illustrative of Kolb’s cycle of reflection and modification of practice. Students valued critical feedback, rather than generic feedback. Some also used it to reflect on their progress and how to plan future learning opportunities. Self directed learning Students used the process of writing up the case to direct their learning. Their techniques reflect experiential and situated learning. By reflection on experience, they transform the medical problem associated with that patient into learning. The fact that it is within the authentic practice of medicine, enhanced and complemented the experience.	*“I was surprised by the amount of feedback that I got…, it was again, just two cases that were reviewed but there was a substantial paragraph with different things. And again, I had like medical related and then content and, sort of, form related feedback. (C3)* *“The feedback was excellent. Much more so than I – I kind of expect, you know, like most uni feedback is like congratulations on submitting your assignment…” (B3)* *“I thought it was really good they included who the markers are, clearly put effort and they clearly read it and (gave) good feedback. I think we all thought it was very surprisingly in depth.” (B7)* *“I was happy she gave me – I think she gave me two really good ones and I was happy with that. Oh, that’s good enough. You know, I can’t ask for more.” (A2)* *“Getting personalised feedback for – for something indicates - -‘cause quite a rigorous exercise is very valuable.” (A1)* *“I think it’s a good idea to get feedback from somebody (an academic) in SMP like that because then it gives us an idea of where we are and where we should be, what we should be aiming for in terms of our final assessments. So I think it is crucial that we do get feedback from them like that. Because…it’s variable, it’s variable depending on which department you’re in, who you are seeing at the time, and the feedback that you get. So I do value the feedback that we get from PebblePad.” (C4)* *“I think because they’re designated people who have stated expectations of what a Sydney medical student long case should be, that was consistent, and…you get…explicit feedback..” (C 3)* *“It’s nice to know what you did wrong so you can do better the next time…Tell us what we’re doing wrong, just about anything…’cause there must always be room for improvement…” “Do feel free to be harsh!” (B5)* *“My first couple were really pretty crap and the feedback I think reflected that but because of the feedback changing it and shifting it….I found that through that process of getting that – that sort of constructive feedback which was very lengthy at first and then gradually got shorter…. it did improve the whole thing, even the verbal presentation too.” (C1)* *“…you get feedback for each one individually, so it was sort of good to – to upload them sort of as I did them every week and then get feedback and then try and use that feedback…for subsequent ones….” (A3)*
Technology and process	
Technical barriers There was general discontent with the technical aspects of the ePortfolio system, Pebblepad. Students had difficulties uploading cases and became frustrated with the duplications of the system. Some students also had trouble finding the feedback, which defeated the purpose of the exercise. These technical aspects detracted from otherwise positive experience of the students.	*“There seemed to be a lot of redundant steps in the software, like having to click things multiple times and then click save and then click submit. Um, the issue I had was I’d submitted about three or four long cases and the clinical school told me I hadn’t submitted any, um, and it turns out I hadn’t clicked the final step.” (C2)* *“I found it a bit cumbersome to use. … the interface..is really difficult, so initially it didn’t actually work on my computer.” (A1)*

### Medical leads’ experience of providing feedback on written long cases and their perception of the value of the learning activity for students

In total, six medical leads were interviewed individually. Five of the medical leads interviewed were female, and one was male. Those interviewed included two General Practitioners, two Endocrinologists, one Haematologist and one Advanced Trainee. Four major themes were identified from analysis of the transcripts: Authenticity of the activity; Feedback and personal development; Ability to track students’ progress; and Technology and process. Results from the interviews are illustrated in Table 3. In line with findings from student focus groups, medical leads indicated that student activities should more closely reflect future career situations; and felt given the depth of written feedback, less iterations of feedback were necessary. Importantly, they felt that the written feedback given was qualitatively different to verbal feedback, providing greater emphasis on structure to the long case. Medical leads identified an increase in self-directed learning by students, who became more pro-active in seeking opportunities for patient interaction, and feedback from clinicians. As noted by Medical leads, the e-portfolio system provided a valuable way to systematically record and track students’ progress.

**Table 3:** Medical leads’ perceptions of their experience of providing feedback on written long cases, and their use of the eportfolio

Authenticity of the activity	
Medical leads indicated they would prefer students to be given a more specific activity that reflects a real working situation for medical practitioners.	*“I think every exercise has to have a point that you’re trying to teach and then we tailor the exercise – rather than say, oh, let’s just make them do a written and a verbal long case but why? What do we want them to learn from* it?” (C 2) *“you present it to someone within 15 min ideally, but by the end of the day you find someone to present it to while it’s fresh, because that will teach them the recall factor, which is what it really is based on. It’s not about writing the perfect story or lecture or admission notes…. I think it’s very important to do it the day they do it…”* (A1)
Feedback and personal development	
Quality of the feedback Medical leads felt that the feedback provided on students’ written work was qualitatively different to that provided on verbal presentations. The written activity with written feedback allowed concentration on correcting the structure of students’ work. Medical leads felt that written feedback provided via the e-portfolio may be more critical, honest and helpful than feedback provided by clinicians while they are busy on wards Quantity of feedback Medical leads felt that less frequent iterations of feedback were required by students Additionally, medical leads felt that if fewer cases were marked, greater consistency in feedback could be provided, which would be valuable for students Self directed learning Medical leads felt the learning activity of performing long cases on the wards, forced students to be pro-active in their clinical placements, assisting students to be less hesitant in approaching patients, and also in approaching clinicians for feedback Medical leads felt the written exercise with feedback helped to develop students’ verbal presentation skills.	*“Written feedback is of course very different and I suppose you have to spend time thinking through how the student has structured their presentation and the structure and the content of the presentation and comment on that. So certainly takes a lot longer… They’re both very useful because you’re assessing different components of the students’ abilities”.* (B2) *“When they were first starting, I would provide very thorough, long feedback, because initially, being new to this, they didn’t know what they were doing, and the structure was all wrong. So I’d actually show them the structure that they should follow, and, clarify what should go in what order, and explain what should be in an opening sentence, tell them if there was too much detail or too little detail, it depended on the student, and then always offer them to come and do one with me early on to get that straight before they went back”*. (A1) *“I think the students need a combination of feedback. So …when you write it.. I would tell them if I think they really need to improve on something. But I can imagine that on the ward, in time pressures, that a lot of people go, “Yeah, yeah, that’s great,” and not actually give them any useful feedback. So I think written, and actually seeing, and, I always give an example of what I meant – so if I said, you know, “You should change it to be more like this,” I’d give them an example of an opening sentence from their case. So they see this is what should be in it. And a lot of people won’t do that if it’s an oral presentation because they are on the run”.* (A1) *“The first lot, by the time I'd marked the long cases they'd already done three…. so the fourth one they tried to change to the way I had suggested in certain things so you could see an improvement …. but often (students uploaded) two or three at a time, at least two at a time”.* (A2) *“Occasionally it would jump around (the structure), of course, depending on the flow. So they should still be able to master that order, and – and arrangement and plan orally, and then just write it down every second one or third one. So I don’t think it needs to be as many”*. (A1) *“You needed to see their progression. And you could see the ones that weren’t progressing.…. in the recent form it was very beneficial to have the same (student) all the time, and then you can tell, yes, you’re getting better at this, this is great, this needs a bit of work. And by the end of it most of them were doing fine for a concise summary of a long case, how you do an admission in the notes”*. (A1) *“I think there’s, um, there is a place for looking at students who are not improving over, say, the first four cases, there’s no sort of signs of improvement. And assigning those people to one person for the next two or three cases”.* (C1) *“I think it’s a good thing because, the more practice you get the better and some students that are struggling or a bit more shy, it sort of forces them to, um, do the cases and get the practice in, um, and it’s certainly very good preparation for their exams*.” (B2) *“Some take very well to immersion training, some are proactive, some become part of the team and engage with patients. There are many who just spend time on the rotation, and would only do things if invited to or if asked to do so. That second group is not insubstantial, they are a fairly large group of students….for a variety of reasons: feel shy, can’t communicate properly, maybe a less of a self-directed learning approach and more of directed learning approach is what they are used to. So for those sorts of students, setting the bar and saying, yes, you have to do one case every week is incredibly usefu*l”. (B2) *“The usefulness of written presentation you could call it, is that it helps the student to develop a structure of where things should go and that in turn, could make, the verbal feedback easier in the long term”*. (B2) *“…the students realise the value of the exercise, of – of having sequential long cases, and – I think the feedback that they’ve given is that it has been a very useful clinical exercise*… *the students have loved it. It’s been very well received. And what it does is, by the time they reached the end of Year 3, they have a sense of what a long case is all about. Which is exactly what this exercise is doing as well. I mean, that – that’s the whole point. So that they don’t hit Year 4 thinking, um, what is a long case?”* (B1)
Ability to track students’ progress	
A major benefit noted by most medical leads was that the e-portfolio system provides a valuable way to systematically record and track students’ progress and identify students needing remediation	*“I had access to everybody’s folders, that meant I could see who had or had not presented and keep an eye on who was lagging behind, so that we could give them a bit of assistance”*. (C1) *“Having the longitudinal collation of cases – that’s a strength. It does work well… something that you do with students who are a bit weaker… you ask them to come back and present to you time and time again. Because if you pick up someone who’s not tracking well…a lot earlier. So I think it is very important… if someone’s just been terribly dismal…. despite feedback, they are not improving… you will often find that that student is weak in other areas too, and then you can bring them in and have a little chat to them about what their study technique or extra remediation exercises or whatever needs to be done. So it gives you a very good index of how – how the student is tracking”.* (B1)
Technology and process	
Technical barriers Technical barriers were encountered by medical leads, who found the ePortfolio system to be cumbersome, and non-intuitive Many of the students were not uploading their long cases consistently, negating the potential benefits of feedback and the opportunity to make improvements Medical leads indicated they would like more interaction with the students to ensure their feedback is received and acted upon	*“I found the system very frustrating - - - - - - and I almost quit because I found it so difficult to use”.* (A2) *If I were to – to describe one major gripe, it’s the swapping of windows that you have to do when you’re editing a student’s work. You can’t sort of edit as you go along. You have to read it separately, and what I’ve been doing is printing it out often, um, reading it through once just to get a sort of flavour, and then going back and giving them feedback. So you just find that it’s quite time-consuming, I think*. (B1) *“There was, a bit of a lag, where students sort of hoarded cases and then suddenly ran out and had to find a whole lot of people to present to…”*(C1) *“The ones who kept on submitting and nothing changed - - - - - - and then I'd be writing, “just keep trying to follow this format” and then they would be – then I think some said, “well, I'd already written them all because I did the long cases 10 weeks ago and I'd already written them” - - And then just put them all up in one. (C2)* *I never really knew if they read them or not read them - - - - or absorbed it or had questions because it, sort of, wasn’t as interactive as potentially the feedback could have been I think.* (A1) *“There were maybe two who wrote back and said, “Yeah, no, that’s good,” and they asked questions and I definitely know that for them it was useful. But then for the – the vast majority who never communicated I don't know how much they took i*n”. (C2)

In summary, our data suggests that students valued the authenticity of the written long case activity. Medical leads felt the task should better reflect a real work situation, with pressure of time in the write-up of the case. Interestingly students perceived the high quality of the feedback contributed to their development as a doctor. Most students felt the incremental benefit from receiving written feedback on more than two of the eight cases was not justified. Of note, there was no difference in the perception of adequacy of feedback between two of clinical schools, A and B. However, students at clinical school C perceived that they received less feedback, and that it was delayed, therefore reducing its value.

While the medical leads felt that feedback provided via the e-portfolio system to be qualitatively different to feedback provided via a face-to-face encounter, it built the capacity of students to understand what is required of them, particularly in terms of the structure of the long case and how to write up a medical admission. Both students and medical leads felt feedback was quite detailed, and that fewer iterations of feedback with greater consistency, at particular time points, would be of greater value to students than more frequent, less detailed feedback.

Major benefits identified by the medical leads were that the long case task increased students’ motivation to engage in clinical activities; and the e-portfolio system provided an efficient method to record and track students’ progress, and identify students in need of remediation. Students and medical leads alike found the ePortfolio system cumbersome. Both parties expressed concern about the detailed steps involved during the process, hindering their active engagement with the feedback. Of concern, some students were not uploading their long cases consistently, and additionally, some medical leads were not providing timely feedback, negating potential benefits.

### Review and analysis of the feedback content provided by the medical leads

Analysis of the feedback, provided by the medical leads, demonstrated in Table 4, identified some key findings to inform future use of the ePortfolio. The feedback was student-focused at all times, with encouragement, even in those who had not performed as well as others, emphasising the concept of continuous professional development and lifelong learning. Authenticity again was a prominent theme with feedback reiterating that the written long case should mimic how notes are written in the real context of hospital practice. Medical leads provided commentary on the difference between verbal reporting and written text on the same case, demonstrating how complementary skills were needed and advice was given on how to develop both skills.

**Table 4:** Feedback provided by medical leads

Personalised, encouraging	You have written up this case really well. Your history and examination are nice and succinct and clear. You have written up this long case thoughtfully and have covered all the required domains. Again you have written up a good case and have thought about the issues in this man. Congratulations on your first long case- you have written this up very well. It is a shame you couldn't examine him, but you have identified what you would look for. Your summary and issues list is great! In the next 4 weeks of medicine, work on investigation and management issues- your histories are excellent; well organised and succinct. Good luck! Another good case- you really write the cases up well- succinct and organised. I am very impressed with your progress Well done on completing your second case! You have improved in your write up from your first.
Authenticity	Only suggestion is think what tests you would do to try and work out cause of fevers.
	Some cases are diagnostic issues and some are management issues- when the diagnosis is clear, then most of your attention should be on management.
	Be careful with abbreviations
	Your issues list is good- think about how you would manage her high urine output. Remember complications of immunosuppression- bones, infections, malignancy.
	You could divide issues into short term, medium term and long term Short term- management peri-operatively of anti-coagulation etc. Medium term- optimisation of CV risk factors, DM etc. Long term- monitoring for complications of DM
	With your issues list, you could probably condense them down to 3 main issues: 1. Rehabilitation following stroke to improve function 2. Manage risk factors including hypertension, hypercholesterolaemia and hyperglycaemia. 3. Poor compliance- importance of ensuring understanding to improve compliance, communication with GP.
	…. writing it up as though you are writing up an admission, succinctly listing the issues, and management plan. For writing the cases up, you should think you are doing an admission and writing it clearly in the notes
Granular and specific	RA history- if possible include the following, particularly if RA is central to the case - When it started? polyarticular vs oligoarticular onset, if known RF, CCP positivity -subsequent course (flares and remission with cumulative deformity for example) -Treatment: prednisone: duration and average dose (if sig steroid side effects) Disease modifying agents- salazopyrin is a weak disease modifying agent, has he ever been on methotrexate or any others? surgical management- eg joint replcements, etc. Current state: active vs inactive, duration of early morning stiffness
	Issues list- just list the issues then move management below 1. Investigation and management of thrombophilia 2. Assess fitness for further surgery 3. Management of multiple co-morbidities- medium to long term 4. Social issues- managing at home and drug/alcohol issues
	Investigations- Standard things, FBC, UEC, LFT, Calcium, cultures if febrile again, etc. How do you monitor if MM in remission??
	Issues list- 1. Control infection, monitor for complications of chemotherapy (infection, plts, anaemia) 2. Complete chemotherapy when well enough- fitness for this? 3. Psychosocial issues- -anxiety (well placed) -Insight into prognosis -carer fatigue
	Issues list- 1. Management of febrile neutropenia 2. Ongoing management of cancer- side effects vs benefits 3. Optimise BP control- particularly in view of aneurysm 4. Socials aspects- isolation/depression/ prognosis and planning for future palliation if required

The main focus of feedback was around getting the structure correct, with examples of how to achieve this rather than “just do it like I want”. Students often narrowly addressed the presenting issue but not other conditions that might be present, and could affect management and long term outcomes. The feedback from medical leads helped to broaden the students’, perspectives, with suggestions on approaches to chronic disease.

In summary the feedback was granular and specific. Medical leads corrected clinical reasoning where needed in case-specific ways, highlighted key features in the history and or examination and incongruences with the written case and final diagnosis/management. At this stage of training, the feedback focused on structure, linking presenting complaints to the differential and problem list to ensure coherence.

## Discussion

Feedback is critical for learning in clinical medicine [10]. This study has provided detailed information about feedback using an ePortfolio system. The main finding is that medical students value feedback that is detailed, personalised and from a clinician with some experience. This is consistent with descriptions of “strong feedback” proposed by Van de Ridder, which included: well observable tasks, expert observer, feedback of highly specific information, personal observation and a plan to re-observe [10]. Other factors, which were described as essential for effective feedback, were that it was timely, constructive and actionable [11]. Credible feedback is often that received from a senior physician who is in a supervisory role [12]. Students in our study identified all these factors as important.

Our students particularly valued critical feedback and specific suggestions on how to improve. They were disparaging of the generic feedback usually provided by the university, such as “satisfactory”, and preferred *“harsh”,* constructive feedback. This is in contrast to the study by Boehler et al. [13], who found that students valued compliments rather than specific feedback on how to improve. In her study she assessed students satisfaction with feedback on surgical knot-tying, with half receiving only compliments and the other half receiving specific, constructive feedback. The main finding was that student satisfaction was higher in the group receiving compliments, whereas the improvement in the task was greater in the group receiving constructive feedback. The conclusion was that student satisfaction was not an accurate measure of quality of feedback, whereas learning was a function of constructive feedback. However, in contrast to Boeler’s study, which focussed on a manual skill, students in this current study were required to complete an authentic and intellectually demanding task.

Triangulation of the data, analysing feedback from the perspective of the teacher and student as well as independent review of the feedback content provided a deeper understanding of the feedback process. Quantity was not seen as important in this study, and most students felt “two was enough”. Analysis of the feedback revealed that high quality and granular initial feedback set the expectations of the student so that subsequent feedback could be very focused and shorter. The perceived benefit of two pieces of personalised, detailed feedback was considered enough, with not much further incremental benefit from more feedback. This certainly is practically important from the point of view of sustainability in providing feedback to approximately 300 students per year.

Details of what is the ideal quantity of feedback have not really been addressed in studies of ePortfolios, assuming that “more was better”. Students using our portfolio system were surprisingly aware and empathetic towards how much time the process of providing feedback was taking clinicians. Additionally, the acceptance and effectiveness of the written feedback appeared to be reliant upon students’ perceived credibility of the person providing the feedback, as has been reported in previous literature [14]. Students felt reassured that their written feedback was provided by a faculty member, familiar with University summative assessment requirements.

The use of an ePortfolio in general facilitated the delivery of feedback but in some cases, because of technical issues, detracted from the feedback process. Some students refused to engage with progressive use of the ePortfolio system, so uploaded all their cases at the end of the block, thus not benefitting from the progressive cycle of reflective practice as described by Kolb [15].

As in other ePortfolios, some students failed to see the purpose of the activity. This is similar to a number of other studies in both medical schools and other health sciences [4, 16]. A few students only appreciated the concept of using the ePortfolio to support the process of learning, to reflect and plan future learning activities. Most saw it as a simple “upload of an assignment”, and did not view the ePortfolio as part of deep learning from feedback.

Although the literature supports the use of an electronic format for providing feedback, previous studies have not looked at the quality and characteristics of the feedback in such detail. In the study of Spickard et al., although the electronic format resulted in more feedback, details of the quality were not explored; the analysis of students’ perception was based on a four-question survey with a five point Likert scale [17]. Belcher et al. [16], describes the use on an ePortfolio in an undergraduate medical program in the UK . Her findings are in contrast with this study in that the students perceived the feedback as either poor-quality or non-existent. This seemed to relate to the delay in provision of feedback and lack of engagement of the supervisors with the feedback process. This differs from two of the clinical schools in our study where the students found the feedback specific, timely and personalised.

Experiential learning and reflective practice are key theories in medical education. In the setting of learning medicine through case-based discussions, such as long cases, students can transform experience into learning [18]. In the medicine ePortfolio, learning was based around real patients, so situated learning complements experiential learning by framing the whole experience in the medical community [19]. The task was authentic which was also important as students could position the task as one required in intern years and beyond. Reflection is also a key aspect of the medicine case history ePortfolio and contributes to the depth of learning; through feedback, the student reflects on this, contextualises it, and improves performance [20].

Another interesting observation was the significant empathy expressed by students towards the medical leads. There was concern about the amount of work required of the medical leads providing feedback to the students. This is in contrast to studies demonstrating a decline in empathy in medical students and residents, perhaps partly due to an inappropriate learning environment, and inadequate role models [21]. A survey of medical students and interns concerning which factors they viewed as affecting empathy during education identified “mentoring and clinical experiences that promote professional growth” to be the most important [22]. It may be that an added benefit of the ePortfolio is providing mentors who can guide development of learning through the ePortfolio.

### Barriers

Technical issues with the Pebblepad platform were the main concern with students using the ePortfolio. Time taken to negotiate and upload cases and difficulty finding feedback were some of the issues raised. This is reflected in the literature regarding ePortfolios. One of the main concerns is the substantial time commitment required by students, with perceived detraction from other learning activities. Time was given as the most important factor limiting the use of the ePortfolio by tutors and students in the study by Duque et al. (2006) [23]. In the study by Belcher et al. [16], students failed to see the purpose of the ePortfolio, feeling that it detracted from clinical time. The findings in this study are similar in that students reported spending too much time engaging with the technical aspects of the portfolio, spending up to 5 h on a single case.

Hall et al. similarly reported their experience with an ePortfolio in Ottawa Medical School and the main barrier to its implementation was the complexity of the system’s design which made its use cumbersome and tedious for students to use [24]. One of the main complaints in this study was the technical aspect of using Pebblepad, which frustrated students and impacted on their engagement with the learning process.

### Limitations of the study

The limitations of this study include issues relating to design and outcome measures. There are seven major metropolitan clinical schools and three rural clinical schools, and the sampling for this study was from three of the major metropolitan schools. These results may not have reflected the experience of feedback of students at other clinical schools or rural sites. It is planned to organise further focus groups at other sites, including rural clinical schools. The sampling should also be more purposive to ensure equal representation of males to female students as it has been shown that males do not necessarily use feedback as a learning experience as well as females [25].

## Conclusions

In conclusion, students valued the feedback provided by the ePortfolio system because of its quality, depth and personalised nature. It was also timely and constructive. However the technical difficulties with the platform had negative impacts on the engagement of some students, and overall the student cohort did not find it “user-friendly”. This study contributes to the literature in outlining the qualities of the feedback which students valued, and that quality was much more important than quantity. The recommendations for further improvements of the system include simplifying the ePortfolio platform, introducing authentic tasks such as discharge summaries and letters to GP and reducing the number of episodes of feedback to two, provided by the same senior clinician, for consistency of feedback. It is also essential that an ePortfolio is integrated both horizontally and vertically across the medical program as a tool for documenting learning, competencies and for reflective practice.

## Additional files

Additional file 1: Interview guide for students. (DOCX 15 kb)
Additional file 2: Interview guide for medical leads. (DOCX 13 kb)
