# Supplementary material for: Feedback using an ePortfolio for medicine long cases: quality not quantity
Source: BMC Med Educ. 2016 Oct 21;16:278. doi: 10.1186/s12909-016-0801-3 (PMC5073895; doi:10.1186/s12909-016-0801-3)
Supplement: Additional file 1: — Interview guide for students. (DOCX 15 kb) [file 12909_2016_801_MOESM1_ESM.docx]

**FEEDBACK USING AN ePORTFOLIO**

**FOCUS GROUP QUESTIONS**

**Student Focus Group Interview schedule**

What has been your experience of using the ePortfolio system?

What aspects have worked well?

Tell me about the feedback you have received through the ePortfolio?

What did you think about the amount and quality of feedback you have received?

How did the feedback you received from the ePortfolio compare to other sources/forms of feedback?

How useful is the feedback you have received?

Did you receive the feedback within a useful time frame?

What do you perceive as the problems with the use of the ePortfolio system?

Do you consider the time taken to complete the task was acceptable?

What changes would make, if possible, to the system or process?
