# Supplementary material for: Feedback using an ePortfolio for medicine long cases: quality not quantity
Source: BMC Med Educ. 2016 Oct 21;16:278. doi: 10.1186/s12909-016-0801-3 (PMC5073895; doi:10.1186/s12909-016-0801-3)
Supplement: Additional file 2: — Interview guide for medical leads. (DOCX 13 kb) [file 12909_2016_801_MOESM2_ESM.docx]

**FEEDBACK USING AN ePORTFOLIO**

**QUALITATIVE AND QUANTITATIVE STUDY**

**Medical Lead Interview schedule**

What has been your experience of using the ePortfolio system?

What aspects have worked well?

Tell me about the type of feedback you provided using the ePortfolio?

What did you think about the process of providing the feedback using the ePortfolio system?

How did the feedback you provided using the ePortfolio compare to other sources/forms of feedback you have given?

What do you perceive as the problems with the use of the ePortfolio system?

Do you consider the time taken to provide the feedback was acceptable?

What changes would make, if possible, to the system or process?

Any other comments you would like to make?

- Do you think the feedback you provided would be useful for students?
- What do you think about the exercise itself, that is students doing one long case per week, face to face?
- Do you think students benefit from both verbal and written feedback?
- What do you think about the quality of the students’ work? Are they where they should be with long cases?
